# Supplementary material for: Dynamic cytoskeletal regulation of cell shape supports resilience of lymphatic endothelium
Source: Nature. 2025 Mar 19;641(8062):465–75. doi: 10.1038/s41586-025-08724-6 (PMC12058511; doi:10.1038/s41586-025-08724-6)
Supplement: Supplementary file 1 — Supplementary Discussion, Methods, Table 1, Figs. 1–7 and References. [file 41586_2025_8724_MOESM1_ESM.pdf]

nature portfolio

https://doi.org/10.1038/s41586-025-08724-6

Supplementary information

Dynamic cytoskeletal regulation of cell shape supports resilience of lymphatic endothelium

In the format provided by the authors and unedited

Supplementary Information

Dynamic cytoskeletal regulation of cell shape supports resilience of lymphatic endothelium

Hans Schoofs[1#], Nina Daubel[1#], Sarah Schnabellehner[1], Max L. B. Grönloh[2], Sebastián Palacios Martínez[3], Aleksi Halme[4], Amanda M. Marks[1], Marie Jeansson[1], Sara Barcos[5], Cord Brakebusch[6], Rui Benedito[7], Britta Engelhardt[5], Dietmar Vestweber[8], Konstantin Gaengel[1], Fabian Linsenmeier[9], Sebastian Schürmann[9], Pipsa Saharinen[4,10], Jaap D. van Buul[2,3,11], Oliver Friedrich[9], Richard S. Smith[12], Mateusz Majda[13], and Taija Mäkinen[1,4,10]*

[1]Department of Immunology, Genetics and Pathology, Uppsala University, Dag Hammarskjölds väg 20, 751 85 Uppsala, Sweden.
[2]Department of Medical Biochemistry at the Amsterdam UMC, location AMC, The Netherlands.
[3]Department of Molecular Cytology, Leeuwenhoek Centre for Advanced Microscopy at Swammerdam Institute for Life Sciences at the University of Amsterdam, The Netherlands.
[4]Translational Cancer Medicine Program and Department of Biochemistry and Developmental Biology, University of Helsinki, Haartmaninkatu 8, 00014 Helsinki, Finland.
[5]Theodor Kocher Institute, University of Bern, Bern, Switzerland.
[6]Biotech Research and Innovation Center, University of Copenhagen, Ole Maaløes Vej 5, 2200 Denmark.
[7]Centro Nacional de Investigaciones Cardiovasculares, Melchor Fernández Almagro 3, E-28029 Madrid, Spain.
[8]Max Planck Institute for Molecular Biomedicine, Münster, Germany.
[9]Institute of Medical Biotechnology, Department of Chemical and Biological Engineering, Friedrich-Alexander University Erlangen-Nürnberg, Paul-Gordan-Str.3, 91052 Erlangen, Germany.
[10]Wihuri Research Institute, Haartmaninkatu 8, 00290 Helsinki, Finland.
[11]Amsterdam UMC, Sanquin Research and Landsteiner Laboratory, The Netherlands.
[12]John Innes Centre, Norwich Research Park, Norwich NR4 7UH, UK.
[13]Department of Plant Molecular Biology, University of Lausanne, CH-1015 Lausanne, Switzerland.

[#]These authors contributed equally.

*Corresponding author:
Taija Mäkinen, E-mail: taija.makinen@igp.uu.se

Content

Supplementary Discussion

Supplementary Methods

Supplementary Tables

Supplementary Table 1. Primary antibodies and reagents used for (immuno)fluorescence

Supplementary Figures 1-7

Supplementary Figure 1. The full scanned images for Extended Data Fig. 9a

Supplementary Figure 2. Characterization of junctional heterogeneity in capillary LECs

Supplementary Figure 3. Whole-vessel reconstruction of a lymphatic capillary perimeter from TEM images

Supplementary Figure 4. Whole-vessel reconstruction of a lymphatic capillary perimeter from TEM images

Supplementary Figure 5. Longitudinal intravital imaging of capillary LEC shape and lobe morphology during homeostasis

Supplementary Figure 6. Generation and characterisation of *R26-LifeAct-EGFP* mice

Supplementary Figure 7. Heart weight and left ventricular morphology and function in *Cdc42* mutant mice

Supplementary References

Supplementary Videos 1-8

Supplementary Video 1. Intravital imaging of capillary LECs showing remodelling of cell-cell borders

Supplementary Video 2. Microtubule anchoring in capillary LECs

Supplementary Videos 3 and 4. Intravital imaging of LifeAct-EGFP showing dynamic actin remodeling in capillary LECs

Supplementary Videos 5 and 5. Intravital imaging of LifeAct-EGFP showing limited actin remodeling in collecting vessel LECs

Supplementary Videos 7 and 8. Time lapse imaging of primary human LECs after optogenetic activation of CDC42

Supplementary Discussion

Silver nitrate staining – background and interpretations. The use of silver nitrate staining for visualizing endothelial cell borders dates back over a century. In this technique, a solution of $AgNO_3$ is typically perfused through an isolated blood vessel, entire organs, or animals. Silver ions in solution bind to argyrophilic substances and upon exposure to light and/or a photographic developer (e.g., 1% NH_4Br, + 3% CoBr solution) these ions will be reduced to microscopic black metallic silver precipitates in a process which requires chloride ions[57,58]. The initial silver foci serve as nucleation sites, where additional silver precipitation occurs, amplifying the intensity of silver deposits over time.

The exact biological entity initiating the silver precipitation at endothelial cell borders has not yet been identified. It has been postulated that basement membrane components, can act as initiators of the silver reaction as they are rich in anionic groups. Previous studies have shown that the basement membrane can be stained at the abluminal side of cell junctions, as observed by TEM analysis[59]. In these earlier experiments, isolated blood vessels were perfused with $AgNO_3$ solution, and cell junctions provided a path of least resistance for silver ions and potentially even metallic silver particles to flow through the intercellular spaces. In our experiments, however, we submerged whole mouse ear dermis into $AgNO_3$ solution, allowing full exposure of the basement membrane to the silver solution. Despite this, we did not observe staining of the basement membrane. Notably, although lymphatic capillaries have a thin, discontinuous basement membrane, collecting vessels possess a relatively thick one. In our staining of collecting vessels, however, we observed clear silver precipitations localized to the cell-cell interfaces, rather than a diffuse, broad staining of the entire vessel surface that would be expected if the basement membrane were the primary initiator of the silver reaction.

Another proposed component responsible for silver precipitation is the so called 'intercellular cement' present at the interface between endothelial cells[2]. The intercellular overlap between capillary LECs is especially wide at sites of intercellular lobes, as shown by our multicolor labeling approach (Fig. 2 g, I, j; Extended Data Fig. 2b), and intercellular cement would be expected to be present at the entire cell-cell overlap. However, we did not observe broad smearing of silver across the LEC overlap but instead fine, segmented lines of silver precipitates, often arranged in a double-

line pattern similar to the organization of VE-cadherin-based junctions, specifically at the borders of capillary LEC lobes. This suggests a more specific interaction at cell-cell junctions rather than a diffuse involvement of the basement membrane or intercellular cement, as suggested previously[60].

Limitations to the study. There are several limitations to our study that should be acknowledged. While we observed shared similarities in cell shape regulation between plant puzzle cells and capillary LECs, there are also notable differences between them. For example, plant epidermal cells are tall cells with rigid cell walls, whereas mammalian LECs are extremely thin, with large overlaps and flexible membranes. Our *in silico* model of mechanical stress on LECs was based on straight cellular interfaces measuring 2 µm in length, and assuming uniform properties throughout, which does not recapitulate the heterogeneity of cell-cell contacts and junctional configurations between LECs *in vivo*. In addition, key parameters such as membrane stiffness, internal cellular pressure, and luminal pressure within dermal lymphatic capillaries *in vivo* remain unknown, which can affect the accuracy of our simulations. Some outcomes of the simulation, such as excessive inflation of cells, may therefore not be physiologically relevant; nevertheless, the model serves to illustrate the effect of cell shape on stress at both the cell and tissue levels. Another difference is that in plants, growth is a stress relaxation process, where cell wall remodelers release some of the tension generated by turgor pressure[61]. In contrast, LECs lack a cell wall, and the mechanism of lobe growth is a cell-intrinsic actin-driven process. Another experimental limitation in our work concerns the isotropic stretching assay, which, like all *in vitro* assays, only partially mimics the *in vivo* context. While it does not recapitulate the full phenotype of capillary LECs including the formation of various intercellular junctions observed *in vivo*, it does promote the key hallmarks of capillary LECs: lobate cell shape and extensive cellular overlaps. These effects are different from those induced by other mechanical forces, such as laminar, oscillatory or transmural flow. For example, isotropic stretch does not promote oscillatory flow-induced changes in the actin stress fibers or pMLC2 levels[62], or transmural flow-induced downregulation of VE-cadherin and jagged adherens junctions[63], which are typically associated with increased paracellular permeability. Further studies should explore the combinatorial role of isotropic stretch with specific external factors, such as flow and growth factors, as well as the composition and stiffness of the extracellular matrix. Finally, our studies focus on dermal lymphatic vessels in mouse ear pinna. Although we obtained evidence of curvilinear and double junctions in lymphatic capillaries in the diaphragm of adult mice, the proportions were not

measured and our analysis was insufficient to determine whether our findings from skin apply to other organs.

Supplementary Methods

Generation of the *R26-LifeAct-EGFP* mice. A conditional knock-in allele was generated by inserting a LifeAct-EGFP (Enhanced Green Fluorescent Protein with a 17 amino acid tag to visualise actin[64]) cassette into the *Rosa26* locus via targeted transgenesis using Recombination-Mediated Cassette Exchange (RMCE) (**Supplementary Fig. 5c**). The transgene contained the following elements: a CAG promoter cassette (containing a cytomegalovirus early enhancer element, a chicken beta-actin promoter and an adenovirus intron) to drive LifeAct-EGFP expression after Cre-mediated recombination; a loxP-flanked transcription termination cassette (STOP) containing a combination of polyadenylation signals (human Growth Hormone and synthetic polyadenylation signals); the LifeAct-EGFP open reading frame[64] together with a Kozak sequence (GCCACC), the human Growth Hormone (hGH) polyadenylation signal and an additional polyadenylation signal. The presence of the NeoR cassette included upstream of the CAG promoter will terminate the transcription from the *Rosa26* promoter. The RMCE vector was transfected into the Taconic Biosciences C57Bl/6ES cell line equipped with RMCE docking sites in the *Rosa26* locus, and the recombinant clones were isolated using Neomycin resistance selection. Mice were generated by Taconic Biosciences. Transgenic mice were detected by PCR with primers designed to amplify a 492 base pair region at the junction of the CAG promoter and the STOP cassette (forward: TGGCAGGCTTGAGATCTGG and reverse: CCCAAGGCACACAAAAAACC). Additional control primers (forward: GGGGCAATCAATTGAGGG and reverse: CAACCTCTGCTTGGTTCTGG) were included in the reaction to amplify a 333 bp fragment. If required, unmodified *Rosa26* allele was detected using primers forward: CTCTTCCCTCGTGATCTGCAACTCC and reverse: CATGTCTTTAATCTACCTCGATGG yielding a 299 bp fragment. Standard amplification reactions (25 µl) were prepared using 0.4 µM of each primer and 0.2 mM dNTPs. After initial denaturation at 95°C for 5 minutes, reactions were subjected to 35 cycles of 95°C (30 s), 60°C (30 s), and 72°C (60 s). Reactions were incubated for a final elongation at 72°C for 10 minutes. PCR products were separated on a 2% agarose gel containing Sybr Safe.

Whole-mount staining of cell surface and total LYVE1. Consecutive staining of surface and total LYVE1 was performed on mouse ear dermis as follows: Samples were fixed for 2 h at room temperature using 4% paraformaldehyde dissolved in PBS, followed by blocking in TBS with 3% bovine serum albumin and 1% fetal bovine serum in the absence of detergents. Staining was performed overnight at 4°C using rat-anti mouse LYVE1 (#MAB2125, R&D Systems) dissolved in

blocking buffer. Tissues were subsequently washed in TBS and incubated with secondary AF647-conjugated donkey anti-rat antibody in blocking buffer. Samples were washed again in TBS and shortly fixed for 20 min at RT in 1% PFA in PBS. To stain for total LYVE1 protein, samples were subsequently permeabilised in TBST (TBS with 0.3% TritonX-100) and blocked in TBST with 3% bovine serum albumin and 1% fetal bovine serum. After incubation with rabbit anti-mouse LYVE1 (#103-PA50AG, Reliatech) and goat anti-mouse PECAM1 (#AF3628, R&D Systems) in blocking buffer, samples were washed in TBST and incubated with secondary donkey anti-mouse AF488 and donkey anti-goat Cy3 antibodies in blocking buffer before further washing and mounting using Mowiol. Similar results were obtained using rabbit anti-mouse LYVE1 (#103-PA50AG, Reliatech) for surface LYVE1 levels and rat-anti mouse LYVE1 (#MAB2125, R&D Systems) for total LYVE1 levels.

Double staining of LYVE1 using *in vivo* injected antibody was performed as follows: rat anti-mouse LYVE1 antibody, conjugated to AF488 (Clone: ALY7, #53-0443-82, Thermo Fisher Scientific) was intradermally injected in a total volume of 20 µl (1:100 dilution in sterile PBS) into the dorsal ear skin of isoflurane-sedated mice. Five minutes later, animals were euthanised by cervical dislocation and ears were immediately dissected and fixed for 2 h at room temperature using 4% paraformaldehyde dissolved in PBS, followed by blocking in TBS with 3% bovine serum albumin and 1% fetal bovine serum in the absence of detergents for 2 h at room temperature. Staining for unpermeabilised LYVE1 protein was performed overnight at 4°C using rabbit anti-mouse LYVE1 (#103-PA50AG, Reliatech) dissolved in blocking buffer. Tissues were subsequently washed in TBS and incubated with secondary AF647-conjugated donkey anti-rat and AF555-conjugated donkey anti-rabbit antibody in blocking buffer for 2 h at room temperature. Samples were washed again in TBS and shortly fixed for 20 min at RT in 1% PFA in PBS. To stain for VE-cadherin protein, samples were subsequently permeabilised in TBST (TBS with 0.3% TritonX-100) and blocked again in TBST with 3% bovine serum albumin and 1% fetal bovine serum for 2 h at room temperature. After incubation with goat anti-mouse VE-cadherin (#AF1002, R&D Systems) in blocking buffer overnight at 4°C, samples were washed in TBST and incubated with secondary AF594-conjugated donkey anti-goat antibodies in blocking buffer for 2 h at room temperature before further washing and mounting using Mowiol.

Confocal microscopy. Confocal images of whole-mount tissues and primary ECs were obtained using a Leica SP8 or Leica Stellaris 5 confocal microscope equipped with 405 nm and white light lasers and

20×/0.75 (HC PL APO CS2), 25×/0.95 HC FLUOTAR L VISIR or 63x/1.3 HC PL APO CORR CS2 Glycerol immersion objectives, and Leica LAS X software. For imaging of the effect of optogenetic activation of CDC42, confocal images were obtained using a Leica SP8 confocal microscope (Leica Microsystems) equipped with a 63× objective (HC PL Apo, C2S, NA 1.40, oil) and a 40× objective (HC PL Apo, CS2, NA 1.30, oil), using unidirectional line scan at a scan speed of 400 Hz and a frame rate of 5.25 s, with 512x512 pixel resolution and 8-bit color depth. The pinhole was set to 2 AU and no averaging was used.

Flow cytometry

Flow cytometry analysis of cycling cells was performed as described previously[65]. Briefly, Ear skin was dissected and digested in 2 ml of digestion buffer containing 5 mg/ml collagenase II (Sigma), 0.2 mg/ml DNaseI (Roche) and 0.2% FBS (Gibco) in 1x PBS for 10 minutes at 37°C and 950 RPM shaking. Digestion was quenched by addition of 10 µl of 0.5M EDTA. Cell suspension was filtered through a nylon 50 µm CellTrics filter (Sysmex) and diluted further to a total volume of 5 ml with FACS buffer containing 0.5% FBS and 2mM EDTA in PBS. Cells were subsequently pelleted by centrifugation and resuspended in 200 µl of fresh FACS buffer. Unspecific binding of antibodies to Fc receptors was prevented by blocking with CD16/32 antibodies (#56603 Biolegend). Endothelial cells were stained using Podoplanin-PE (#127410, Biolegend) and CD31-Pe-Cy7 (#102418, 444 Biolegend). Immune cells and dead cells were excluded by staining with CD45-eF450 (#48-0451-82, ThermoFisher), CD11b-eF450 (#48-0112-82, ThermoFisher) and Ter119-eF450 (#48-5921-8, ThermoFisher) antibodies. After antibody incubation, cells were washed using 1xPBS and dead cells were stained using blue LIVE/DEAD® fixable dead cell stain kit (Life Technologies). Cells were subsequently fixed and permeabilised using Foxp3/Transcription factor staining kit (eBioscience) following manufacturer's instructions and stained for Ki67-e660 (eBioscience, 50-569). Cells were analysed on BD LSRFortessaTM Cell Analyzer (BD Biosciences) using BD FACSDiva Software (Version 8.0) (BD Biosciences). Results were further processed using FlowJo software (version 10.5.0-10.5.3) (TreeStar).

Image quantification. *Junctional classification.* Analysis of junction morphology was done on blunt-ended initial lymphatic capillaries in the segment between the initial tip and the first valve. Junction types were quantified in Z-stack projection by numbering of individual lobes of LYVE1 and VE-cadherin-stained LECs and subsequent categorizing of lobe-associated junctions based on

VE-cadherin signal. Five categories were defined: 1) *Button junction* – a punctate VE-cadherin$^+$ deposit at the neck of LYVE1$^+$ lobe/overlap, with no detectable VE-cadherin at the borders of or within the LYVE1$^+$ overlap, 2) *Curvilinear junction* – unsegmented or segmented distribution of VE-cadherin within one border of LYVE1$^+$ lobe/cellular overlap, 3) *Double junction* – unsegmented or segmented distribution of VE-cadherin within both borders of LYVE1$^+$ lobe/cellular overlap, 4) LYVE1$^-$ curvilinear junction – unsegmented linear VE-cadherin distribution at cell-cell contacts in the absence of LYVE1, and 5) *Zipper junction* – continuous linear VE-cadherin distribution surrounding the entire cell in the absence of LYVE1. Wild-type C57BL/6J mice were used for analysis of junction types, and 4-5 blunt ended vessels per mouse from five mice per age group and condition were analysed; in total 1785 junctions were annotated (available in Zenodo with the identifier doi: 10.5281/zenodo.13880404). Individual datapoints are presented as weighted averages per mouse, to allow presenting conclusions on junctional frequencies at the level of the individual capillary analyzed, while at the same time being able to accurately report on individual mice.

Microtubules. Microtubule anchoring was assessed by manually drawing the cell outlines of LYVE1, VE-cadherin and alpha-tubulin stained LECs and subsequent marking of contact points of microtubule ends with the cell outline by going through the individual optical slices. Contact points were manually deemed as associated with either a concave or convex part of the cell outline and their respective ratio calculated. Microtubule density was obtained by dividing the cell outline into convex and concave parts and measuring their respective length. Then, the number of microtubule ends in contact with the cell outline at each respective concave or convex area was calculated and represented as a fraction of the length of either convex or concave areas. Microtubule analysis was carried out using *LifeAct-EGFP;Vegfr3-CreER^{T2}* as well as heterozygous littermate control *Cdc42$^{flox/+;}$LifeAct-EGFP;Prox1-CreER^{T2}* mice, n=5 LECs from 2 mice, 8-29 MT/cell (Ctrl 9 wk); n=5 LECs from 2 mice, 26-39 MT/cell (Mut 9 wk); n=5 LECs from 3 mice, 12-36 MT/cell (Ctrl 12 wk); n=7 LECs from 2 mice, 22-61 MT/cell (Mut 12 wk).

TEM data. Quantification of TEM data was carried out in FIJI/ImageJ using TEM images of cell-cell overlaps in cross sections of lymphatic vessels. Length of cellular overlaps was measured as the length of direct contact areas between two cells. Lengths of discontinuous contact areas within one cellular overlap were added. Manual counting of electron dense junctions within cellular overlaps was done in Adobe illustrator.

Cellular overlaps. Quantification of cellular overlaps in the *iMb2-Mosaic*;*Vegfr3-CreER*^{T2} mice was done in Fiji/ImageJ. A maximum projection was obtained from the z-stack images, and single channels representing individual fluorophores were thresholded using the default algorithm to obtain a binary image. Binary thresholding was improved using both erode and dilate filters to remove individual pixels and fill gaps in the binary cell surface, respectively. A mask was created using the binary image and overlayed on the original image to confirm accuracy of the thresholding. Binary images of individual neighbouring cells expressing different reporters were overlayed and pixels present in both channels (overlap) were duplicated into a new image. Area of overlap was calculated and divided by the entire length of the overlap to obtain relative width of the cell-cell contact area. n=26 overlaps from 4 mice (Ctrl uninjected), n=35 overlaps from 4 mice (PBS injected), n=25 overlaps from 4 mice (*Cdc42*$^{flox/+;}$*R26-iMb2;Prox1-CreER*^{T2}), n=30 overlaps from 4 mice (*Cdc42*$^{flox/flox;}$*R26-iMb2;Prox1-CreER*^{T2}), n=5 overlaps from 2 mice (collecting vessels).

Cell and vessel parameters. Quantification of cell area in the *iMb2-Mosaic*;*Vegfr3-CreER*^{T2} mice was done using maximum projection images of labelled LECs, which were individually thresholded, and surface area was calculated using ImageJ. To verify accuracy of this method, some cells (**Fig. 2d**, 9-week-old timepoint, 5 datapoints) were processed using Imaris (v9) (Bitplane) by surface rendering and comparable cell surface areas were obtained. Quantification of capillary diameter was performed automatically and in a blinded manner using REAVER MATLAB script for vessel analysis[66]. Number of lobes was counted manually by two researchers on individual images of cells labeled with *R26-iMb2* fluorophores using Fiji/ImageJ.

Primary EC analyses. Quantification of cellular overlaps (HDLECs or HUVECs) and acellular area (HDLECs) in cultured primary cell monolayers was done in Fiji/ImageJ. PECAM1$^+$ cellular overlap area was quantified using the REAVER MATLAB script[66] and manually curated to improve accuracy of the automated analysis using maximum intensity projection images (minimum dimension of 54 000 µm^2, for HDLECs n=13 unstretched Ctrl, n=19 stretched Ctrl, n=12 unstretched + ML141, n=16 stretched + ML141, from 3 independent experiments, for HUVECs n=4 unstretched, n=6 stretched, for mAb13 experiments n=23 unstretched Ctrl, n=13 unstretched + 0.1 µg/ml mAb13, n=12 unstretched + 0.2 µg/ml mAb13, n=24 stretched Ctrl, n=14 stretched + 0.1 µg/ml mAb13, n=15 stretched + 0.1 µg/ml mAb13, from three independent experiments). The overlap area was divided

by the total junction length in each image, obtained after segmentation and skeletonization of linear cell-cell junctions (VE-cadherin staining) and normalised to the average overlap width in unstretched controls in each experiment. The corresponding segmentation was performed by thresholding using the default algorithm and binary images created using both the *close* and *dilate filter* function to remove artifacts from wider and irregular shaped junction areas, as well as individual pixels. For quantification of intercellular separations, maximum intensity projection images of VE-cadherin staining (minimum dimension of 0.38 mm^2, n=32 unstretched Ctrl (including untransfected + siScramble), n=35 stretched Ctrl (including untransfected + siScramble), n=6 unstretched + ML141, n=8 stretched + ML141, n=24 unstretched + siCDC42, n=29 stretched + siCDC42, from 3 (ML141) or 4 (siRNA) independent experiments) were inverted, thresholded using the default algorithm and binary images were created using the *close filter* function to remove individual pixels. Further removal of individual pixels was performed by using *noise, despeckle* function. To confirm the accuracy of the binary image, a mask was created and overlayed onto the original maximum intensity projection of the VE-cadherin channel. The area of intercellular separations was normalised to total image area (% acellular area). Cell-cell junction curvature was determined in segments between multicellular contacts points using the junction linearity index[67], by measuring the ratio of “junction contour” to “straight line junction length” in Fiji/ImageJ, n=8 images with 4-9 junctions/image from 4 chambers (unstretched) and n=6 images with 6-7 junctions/image from 3 chambers (stretched). The active integrin β1 (clone HUTS-4) positive area percentage was quantified in Fiji/ImageJ. First, the filter Gaussian blur (Sigma (radius) = 4) was applied and the background subtracted (Rolling ball radius = 50 pixel). Next, binary images were created by thresholding all images with the same parameters and the resulting area percentage was quantified per image (n = 4-6 images from each condition from one experiment).

Tracer clearance. Quantification of tracer clearance was performed in Fiji/ImageJ on maximum intensity projection of whole ears. A ROI was drawn above the injection site at t0, and the sum of the pixel intensities within the ROI was calculated using the raw integrated density (RawIntDen) function. The same ROI was applied to the maximum intensity projection image 4 hours after injection, and the sum of the pixel intensities was measured again. Clearance rate was calculated by dividing $RawIntDen^{t4hours}$ by $RawIntDen^{t0hours}$ and inverting the result to obtain cleared fluorescence. n=8 mice (*Cdc42^{flox/flox}*), n=6 mice (*Cdc42^{flox/flox};Prox1-CreER^{T2}*).

Line intensity scans. Intensity plots of immunofluorescence staining were generated in Fiji/ImageJ and plotted in Graphpad Prism (version 9). A rectangular ROI was drawn over the line of interest and average intensity for each channel across the length of the rectangle was measured using intensity profile function. Data were extracted and plotted in Prism whereby each channels has its own free axis.

Parallelised isotropic cell stretch device. Developed as an extension of our *IsoStretcher* system[68], this mechanically actuated system enables a simultaneous in-plane isotropic stretch of four custom-moulded PDMS chambers in parallel. The new system was redesigned to have force transmission changed from a one-step V-belt gear to a three-staged cogwheel gear. In this latter concept, the rotation of a stepper motor is transferred over three stages of cogwheels to the PDMS chambers. The last stage consists of 24 cogwheels with vertical pins attached at the top and arranged in four hexagonally ordered mounting spots, where the PDMS chambers are mounted onto the vertical pins. Thus, the motion of the motor is equally split between all four PDMS chambers. The cell stretching device is designed to apply isotropic stretch of up to ~20% in diameter expansion of the PDMS chambers, which equals about ~40% area enlargement of the stretching membrane. Stretch cycle length is gradually adjustable. The device is controlled via a custom-made software to execute the stretch experiments with the requested settings. Details of the *MultiStretcher* device will be presented elsewhere (FL, SS and OF, in preparation).

Western blot analysis. HDLECs were lysed in RIPA buffer, supplemented with 50 nM Na_3VO_3, PhosSTOP™ phosphatase inhibitor cocktail (4906837001, Roche) and cOmplete™ protease inhibitor cocktail (4693116001, Roche). The denatured protein lysates were separated by sodium dodecyl sulfate polyacrylamide gel electrophoresis (SDS-PAGE) on 4-12% Bis-Tris gradient gels (NP0335BOX, Invitrogen) using the NuPage™ MOPS SDS running buffer (NP0001, Invitrogen), followed by transfer onto polyvinylidene difluoride (PVDF) membranes (Immobilon-P, Millipore) using the NuPage® transfer buffer (NP0006-1, Invitrogen). The primary antibodies rabbit anti-human CDC42 (2466S, Cell Signaling) and rabbit anti-human GAPDH (2118, Clone14C10, Cell Signaling) were used for protein detection. Western blots were developed using donkey anti-rat HRP (711 035 152, Jackson) and the ECL™ Prime western blotting detection reagents (RPN2232, Amersham). Uncropped gel images can be found in Supplementary Fig. 1.

Echocardiography. *Cdc42*$^{flox/flox}$*;Prox1-CreER*^{T2} and control (*Cdc42*$^{flox/flox}$ or *Prox1-CreER*$^{T2+}$) mice were received three consecutive administrations of 1 mg tamoxifen in oil via oral gavage. Three weeks later, standard 2D echocardiographic study was performed as described[69], using a Vevo 2100 imaging system and 22-55 MHz echocardiography transducer (MS550D; Vevo2100, FUJIFILM VisualSonics, Canada). Mice were anesthetised with 2% isoflurane in oxygen at 1 L/minute and maintained with a nose cone on a heated stage in the supine position. Fur was removed from the chest using a depilatory cream. For analysis of left ventricular (LV) dimensions, images were acquired in parasternal long axis view. Subsequent images were analysed using 2D quantifications on Vevo 2100 software taking an average of 4 measurements per variable. Following echocardiography, animals were sacrificed, and the heart was removed, weighed and normalized to total body weight.

Supplementary Table 1. Antibodies and reagents used for (immuno)fluorescence.

**Primary antibody**	**Provider and catalog number**	**Dilution**
Chicken anti-GFP	Abcam, ab13970	1:200
Goat anti-mouse VE-cadherin	R&D Systems, AF1002	1:200
Goat anti-mouse PECAM1	R&D Systems AF3628	1:200
Goat anti-mouse VEGFR3	R&D Systems, AF743	1:100
Hamster anti-mouse PDPN (8.1.1, PE)	eBioscience, 12-5381	1:300
Mouse anti-HA tag, Alexa Fluor 647	Cell Signalling Technology, 6E2	1:100
Mouse anti-human PECAM1, clone JC70A	Dako, M0823	1:150
Mouse Anti-Integrin β1 Antibody, activated, clone HUTS-4	EMD Millipore, MAB2079Z	1:200
Mouse Integrin β1/ITGB1 Antibody (TS2/16), Alexa Fluor 488	Santa Cruz, sc-53711 AF488	1:100
Rabbit anti-alpha tubulin	Abcam, ab52866	1:50
Rabbit anti-GFP	ThermoFisher A-11122	1:500
Rabbit anti-DsRed	Takara Bio, 632496	1:200
Rabbit anti-mouse LYVE1	Reliatech, 103-PA50AG	1:200
Rabbit anti-mouse CLDN5	Invitrogen, 34-1600	1:100
Rabbit anti-CDC42, Clone 11A11	Cell Signalling Technology, 2466	1:500
Rabbit anti-human GAPDH, Clone 14C10	Cell Signalling Technology, 2118	1:2500
Rabbit Phospho-Myosin Light Chain 2 (Ser19)	Cell Signalling Technology, 3671	1:200
Rat anti-mouse CD11b (M1/70, eF450)	eBioscience, 48-0112	1:50
Rat anti-mouse CD16/CD32	eBioscience, 14-0161	1:100
Rat anti-mouse CD45 (30-F11, eF450)	eBioscience, 48-0451	1:50
Rat anti-mouse TER-119 (TER119, eF450)	eBioscience, 48-5921	1:100
Rat anti-mouse Ki67 (SolA15, eFluor 660)	eBioscience, 50-5698	1:100
Rat anti-mouse LYVE1	R&D Systems, MAB2125	1:200
Rat anti-mouse PECAM1	BD Pharmingen, 553370	1:200
Rat anti-mouse PECAM1/CD31 (390, PE-Cyanine7)	eBioscience, 25-0311	1:300
Rat Anti-human CD29, Clone Mab 13	BD Pharmingen, 552828	0.1-0.2 µg/ml
Rat Anti-mouse CD29, Clone 9EG7 (RUO)	BD Pharmingen, 553715	1:100
Rat anti-mouse LYVE1- Alexa Fluor™ 488, Clone ALY7	Invitrogen, 53-0443-82	1:100

**Secondary antibody**	**Provider and catalog number**	**Dilution**
Donkey anti chicken IgY-AF488	JIR, 703-545-155	1:500
Donkey anti-rat IgG-Cy3	JIR, 712-165-153	1:500
Donkey ant-rat IgG-Dylight 405	JIR, 712-475-153	1:500
donkey anti-rat IgG-AF488	JIR, 712-545-153	1:500
donkey anti-rat IgG-AF647	JIR, 712-605-153	1:500
donkey anti-goat-AF647	JIR, 705-605-147	1:500
donkey anti-goat-AF594	JIR, 705-585-147	1:500
donkey anti-goat-AF680	JIR, 705-625-147	1:500

donkey anti-rabbit-AF488	JIR, 711-545-152	1:500
donkey anti-rabbit-A647	JIR, 711-605-152	1:500
Donkey anti-rabbit-Cy3	JIR, 711-166-152	1:500
donkey anti-rat IgG-AF405+	ThermoFisher, # A48268	1:500
donkey anti-rat IgG-AF488+	ThermoFisher, # A48269	1:500
donkey anti-rabbit IgG-AF555+	ThermoFisher, # A32794	1:500
donkey anti-goat IgG-AF647+	ThermoFisher, # A32849	1:500

JIR, Jackson ImmunoResearch.

**Other**	**Provider and catalog number**	**Dilution**
DAPI	Sigma Aldrich, MBD0015-1ML	1:1000
SPY-555 actin (1000X)	Spirochrome	1:1000

Supplementary Figures

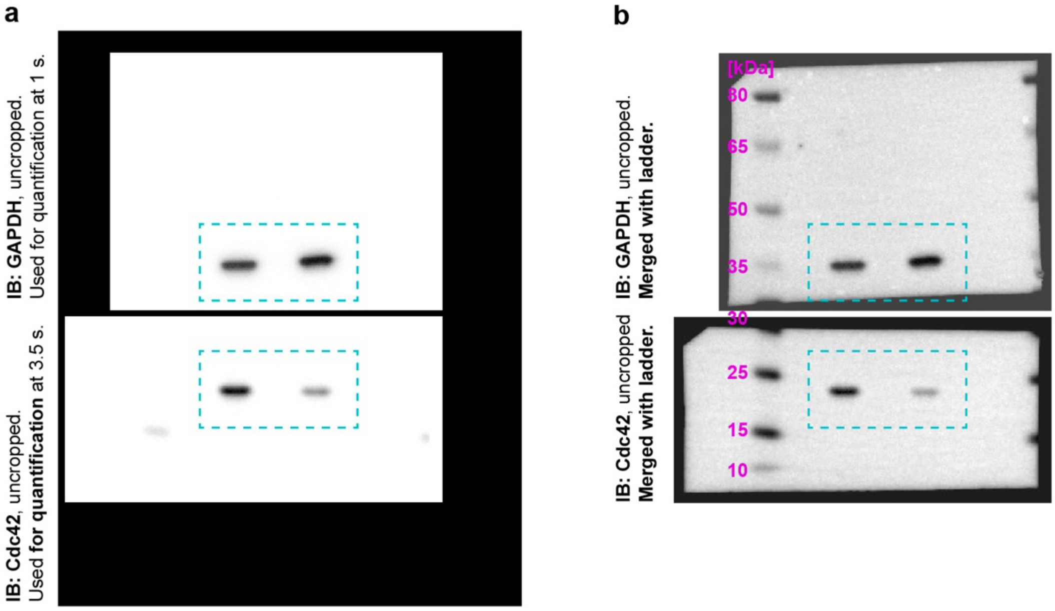

Supplementary Figure 1. The full scanned images for Extended Data Fig. 9a. Uncropped membranes used for images and quantification (**a**), and dislayed as merged with ladder (colorimetric) signal (**b**). Cropped areas are indicated by dashed box, molecular weights (kDa) are indicated in magenta. PVDF membrane was cut horrizontally into two pieces at 30 kDa. Membrane width was independently cut to fit the staining containers. The upper part (>30 kDa) was blotted using antibodies against GAPDH and the lower part (<30 kDa) against CDC42. Cropped areas indicated by dashed box. Molecular weights (kDa) in magenta.

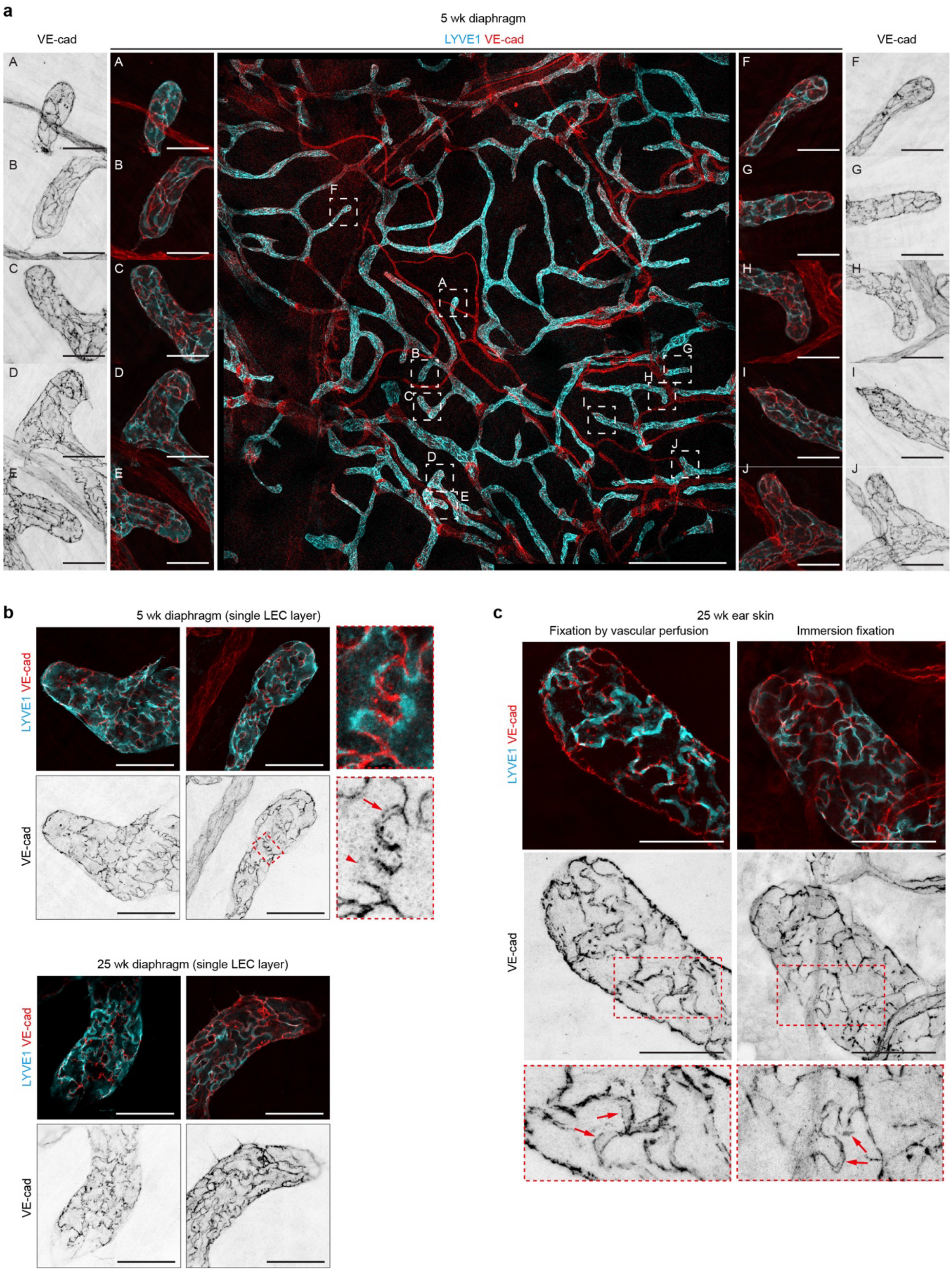

Supplementary Figure 2. Characterization of junctional heterogeneity in capillary LECs. Whole-mount immunofluorescence of lymphatic vessels in the diaphragm (**a, b**) or ear skin (**c**) of 5- or 25-week-old mice. Boxed areas are magnified as indicated. Images in (**b**) represent a sub-stack across a single LEC layer. In (**c**), tissue was fixed by vascular perfusion (left) or immersion after dissection (right). Arrowhead in (**b**) indicates a button junction, arrowheads in (**b, c**) double junctions. Scale bar: 500 µm (a, overview), 50 µm (a, magnifications, b, c).

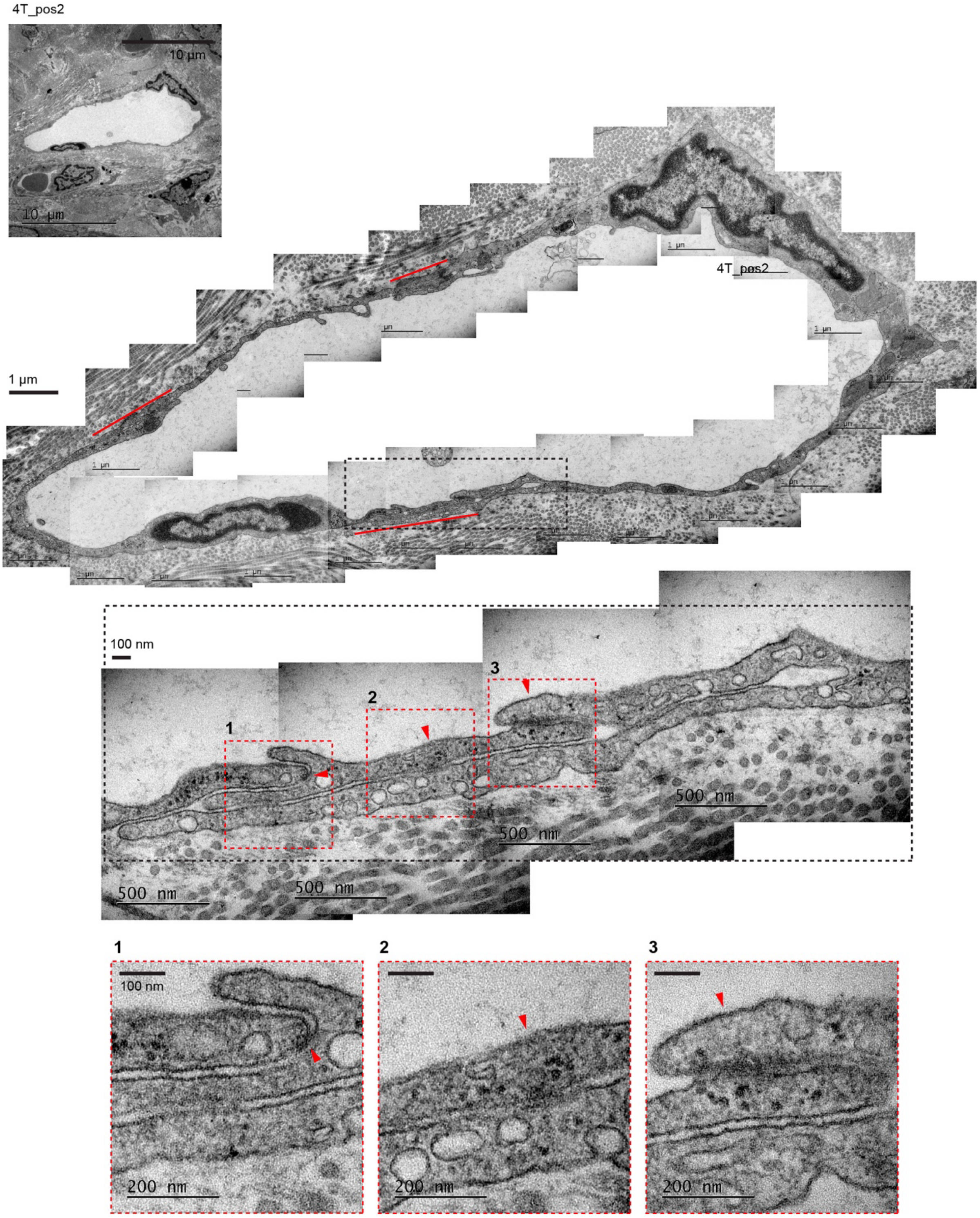

Supplementary Figure 3. Whole-vessel reconstruction of a lymphatic capillary perimeter from TEM images. Cellular overlaps are indicated by red lines, electron densities indicating junctional components by red arrowheads. Boxed areas (dotted lines) are magnified. Note high variability in the length of cellular overlaps, likely related to the sectioning angle.

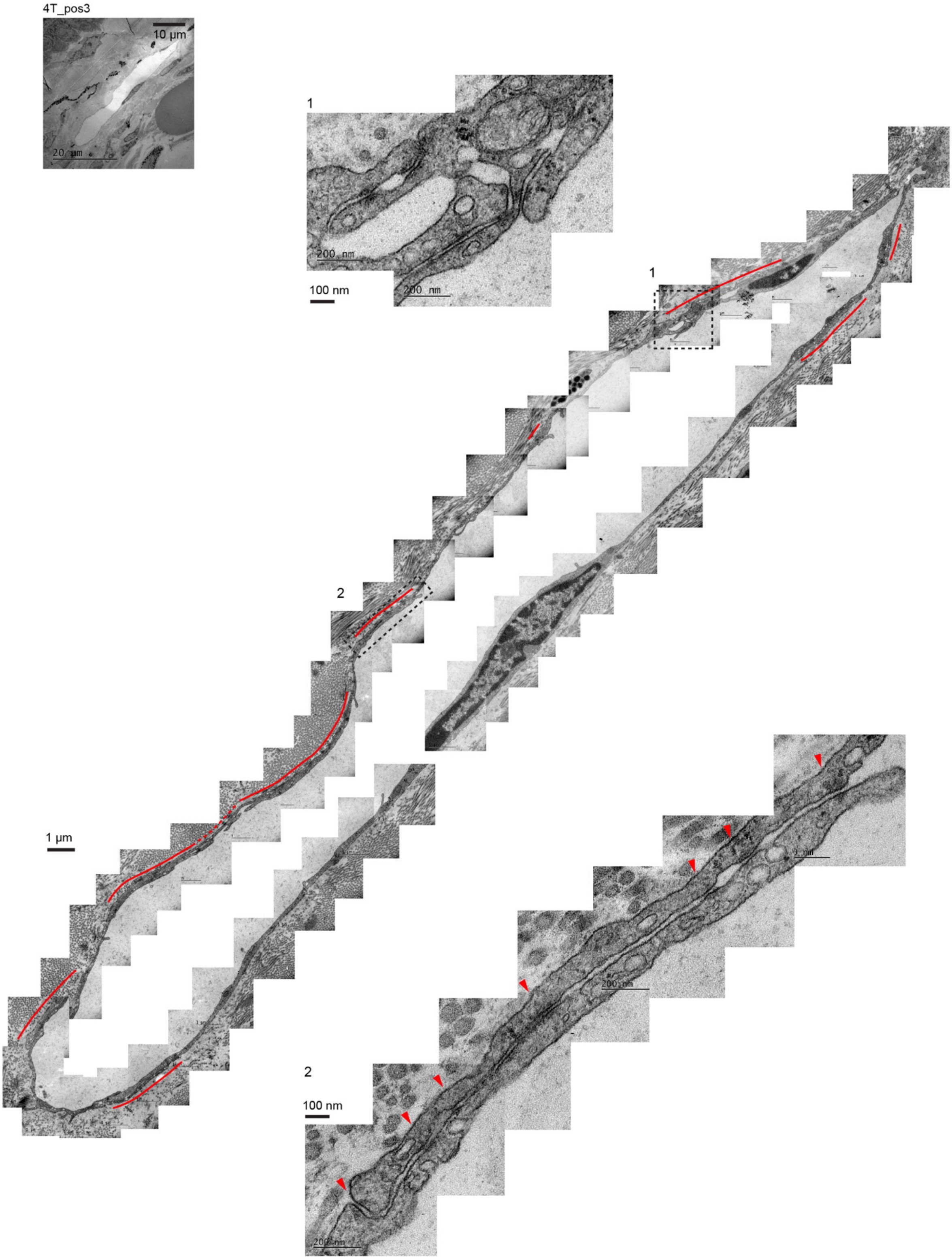

Supplementary Figure 4. Whole-vessel reconstruction of a lymphatic capillary perimeter from TEM images. Cellular overlaps are indicated by red lines, electron densities indicating junctional components by red arrowheads. Boxed areas (dotted lines) are magnified. Note high variability in the length of cellular overlaps, likely related to the sectioning angle.

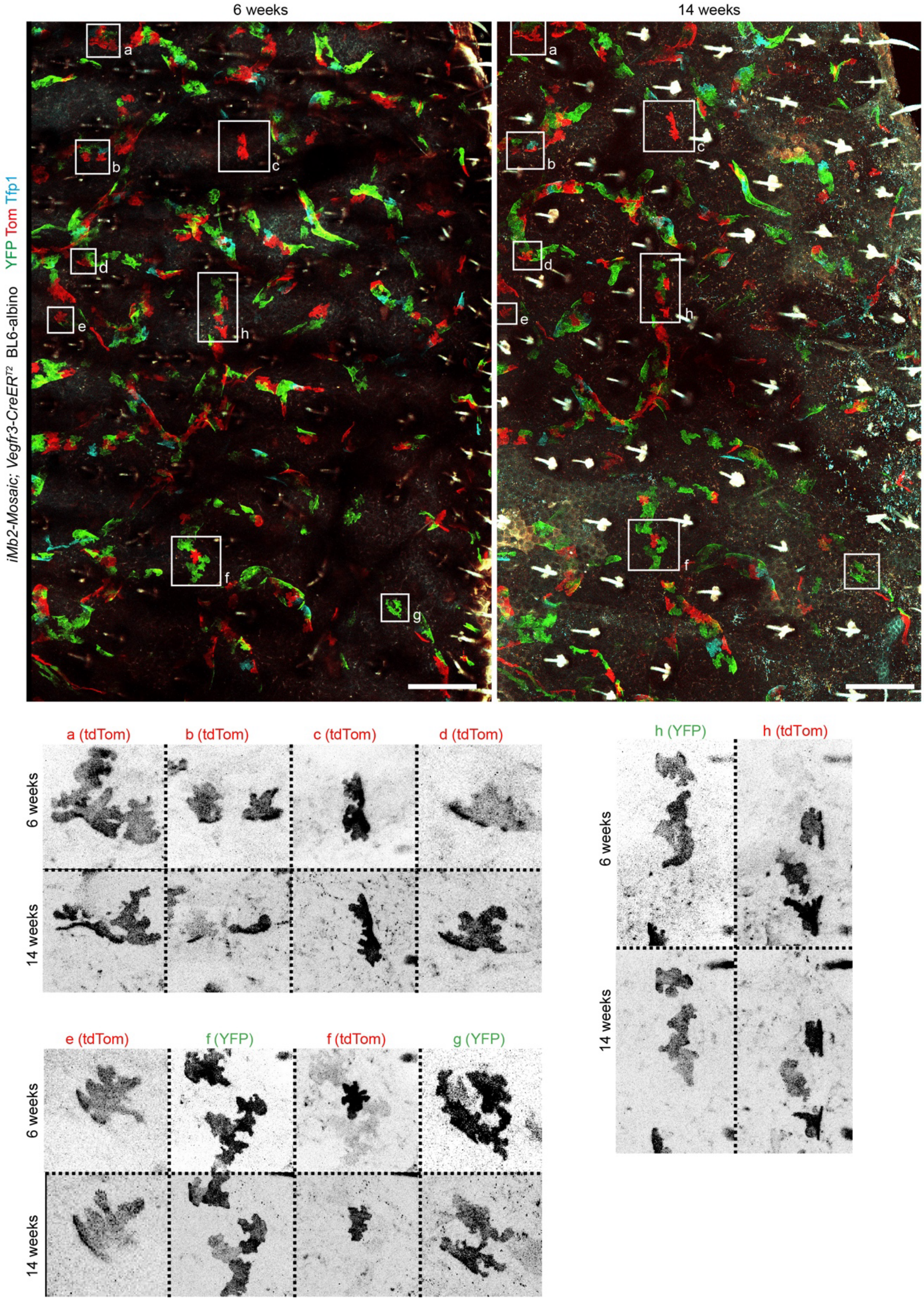

Supplementary Figure 5. Longitudinal intravital imaging of capillary LEC shape and lobe morphology during homeostasis. Tile scan overviews of intravitally imaged capillary LECs in an *iMb2-Mosaic;Vegfr3-CreER*^{T2} BL6-albino mouse using 2-photon microscopy showing remodeling of LEC lobes during an 8-week period between the first (6 weeks of age) and last (14 weeks of age) imaging session. Areas annotated in top panels are magnified in lower panels. Scale bar: 200 µm.

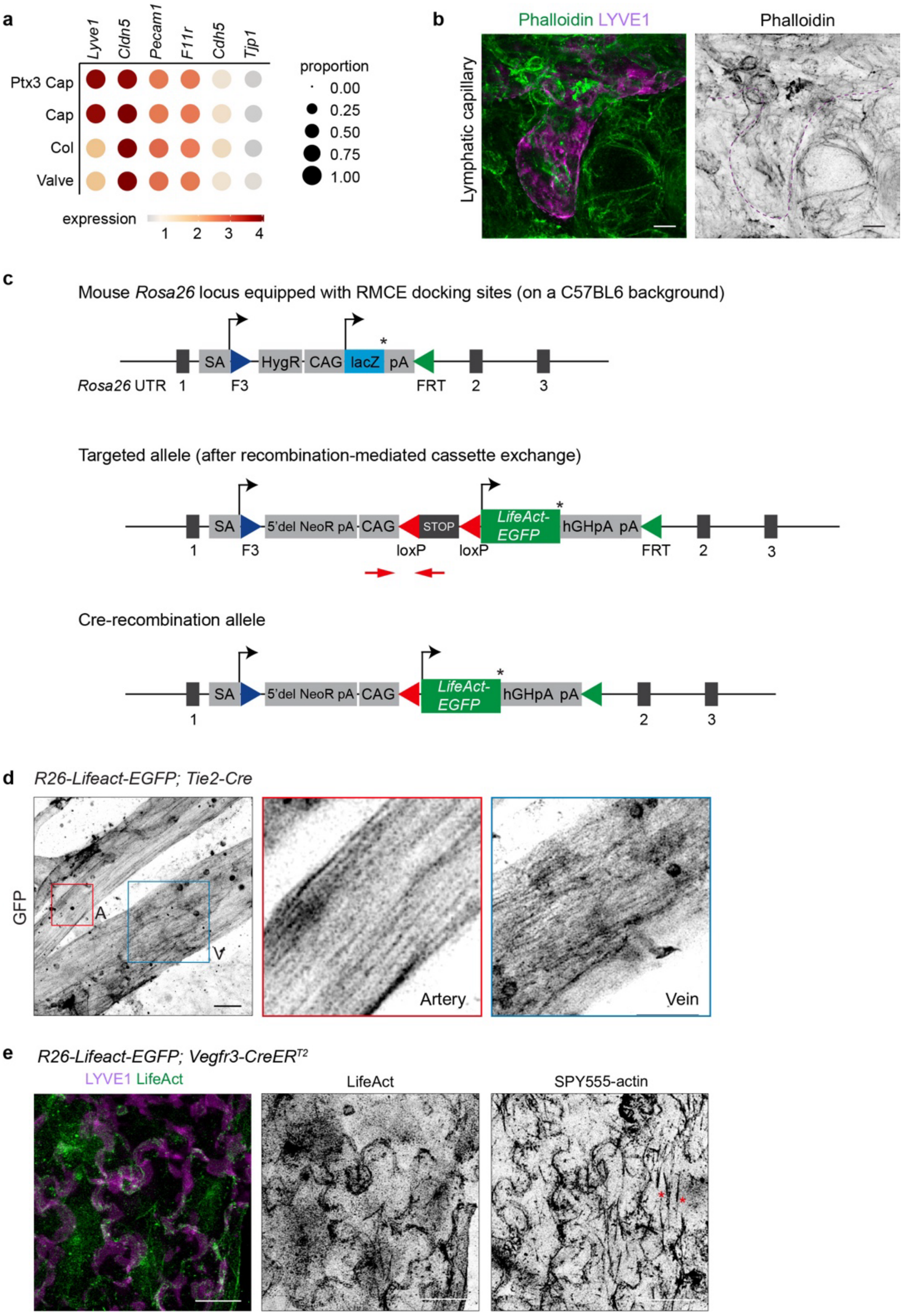

Supplementary Figure 6. Generation and characterisation of *R26-LifeAct-EGFP* mice. (**a**) Dot plot of expression levels of major junctional adhesion components showing similar expression levels in capillary and collecting vessel LECs. Dot size illustrates percentage of cells with transcript sequence counts, and color illustrates the average expression level (log2 fold change) within a population. (**b**) Whole mount phalloidin staining of an adult mouse ear skin (corresponding to **Fig. 3e**), showing no discernible signal in LECs due to high F-actin levels in cells within the surrounding tissues. (**c**) Schematic of the genomic locus equipped with docking sites for the Recombination-Mediated Cassette Exchange (RMCE) targeting vector, as well as the targeted allele before and after Cre-mediated recombination. SA, splice acceptor site; hGH, human growth hormone; pA, polyadenylation site; STOP, transcription termination cassette; CAG, CAG promoter. The genotyping primer locations are indicated by red arrows. (**d**) Whole-mount immunofluorescence of ear skin

from a 3-week-old *LifeAct-EGFP;Tie2-Cre* mouse showing actin stress fibers in arterial (A) and venous (V) ECs. (**e**) Visualization of actin cytoskeleton in LECs in the ear skin of a tamoxifen-treated adult *LifeAct-EGFP*;*Vegfr3-CreER*^{T2} mouse. Tamoxifen was administered at 3 weeks and ears analyzed at 9 weeks of age. Co-staining with the actin dye SPY-555 shows actin accumulation at LEC borders. Red asterisks indicate signal from surrounding cells. Scale bar: 10 µm (d, e).

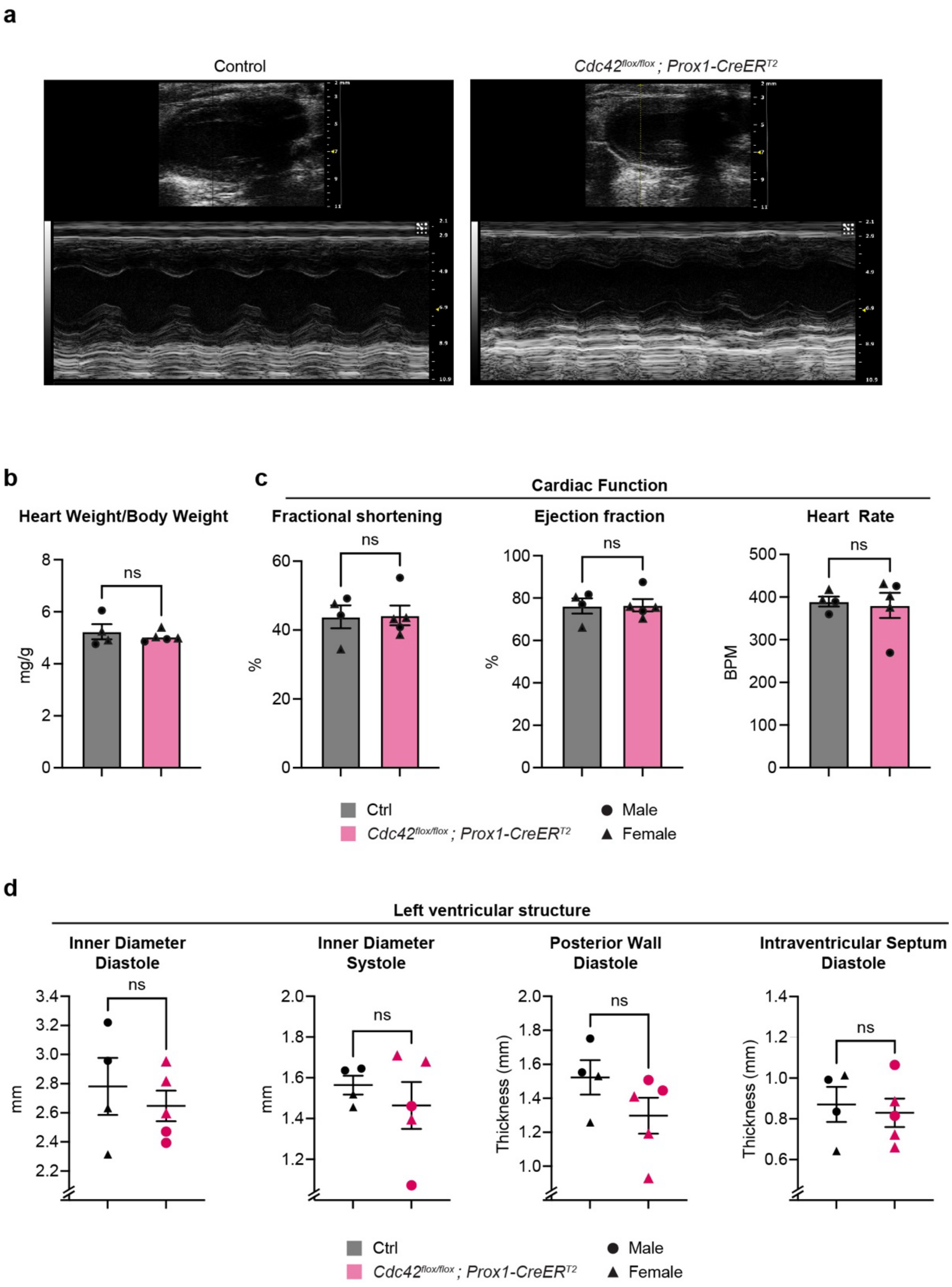

Supplementary Figure 7. Heart weight and left ventricular morphology and function in *Cdc42* mutant mice. Echocardiography of left ventricular structure and function visualized using parasternal long axis view in M-mode of *Cdc42*$^{flox/flox}$;*Prox1-CreER*^{T2} (n=5) and control mice (*Cdc42*$^{flox/flox}$ (n=2) and *Prox1-CreER*$^{T2+}$ (n=2)), 3 weeks after tamoxifen treatment (a). No differences between the groups were observed in heart weight/body weight (b), cardiac function parameters (fractional shortening, ejection fraction and heart rate) (c), and left ventricular structure (inner diameter, posterior wall thickness and intraventricular wall thickness) (d). Data are presented as mean ± SEM; ns, not significant ($p > 0.05$). Two-sided Mann Whitney U test.

Supplementary Videos

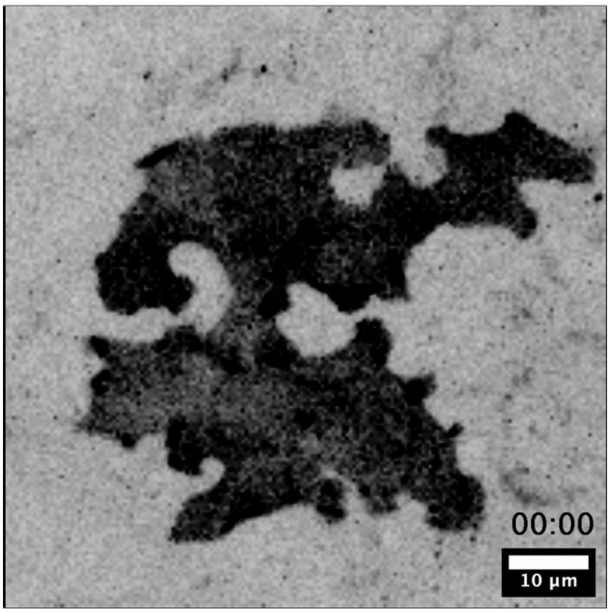

Supplementary Video 1. Intravital imaging of capillary LECs showing remodelling of cell-cell borders. Intravital imaging using two-photon microscopy of individual YFP-expressing LECs in intact ear skin dermis of *iMb2-Mosaic*;*Vegfr3-CreER*^{T2} BL6-albino mice. Note remodelling at capillary LEC lobes but not in concave regions of the cell. Maximum projection of Z-stacks. Scale bar: 10 µm, total length 210 minutes, speed 5:00 min:sec/frame.

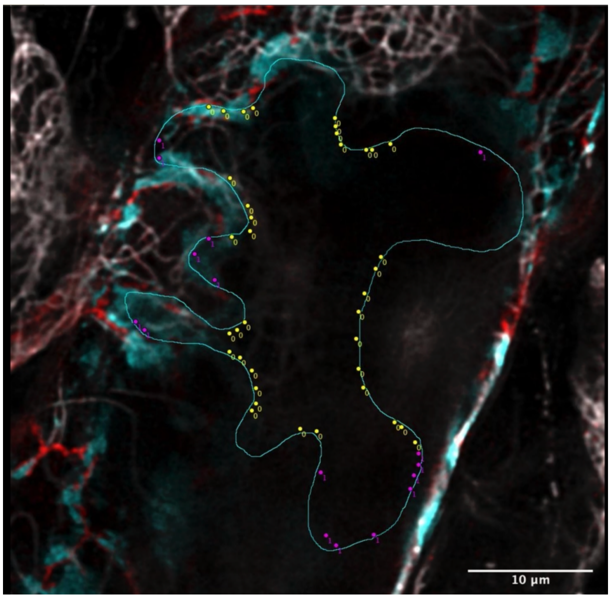

Supplementary Video 2. Microtubule anchoring in capillary LECs. Video of single slices of Z-stack shown in **Fig. 3b, c.** Whole-mount immunofluorescence of adult ear skin stained for LYVE1 (cyan), VE-cadherin (red) and alpha-tubulin (grey). Cell outline is shown in cyan, concave microtubule anchor points are annotated in yellow (0), convex anchor points in magenta (1). Scale bar: 10 µm

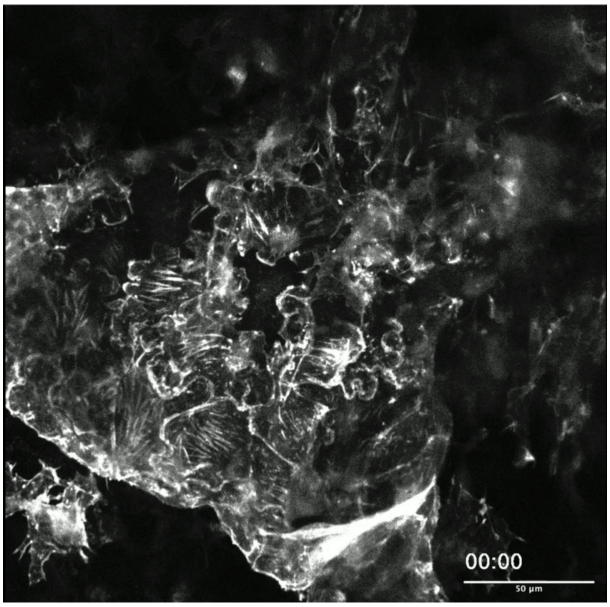

Supplementary Video 3. Intravital imaging of Lifeact-EGFP showing dynamic actin remodeling in capillary LECs. Real-time intravital two-photon imaging of a dermal lymphatic capillary in a 9-week-old *Cdc42*$^{flox/+}$*; LifeAct-EGFP; Prox1-CreER*^{T2} mouse. Labelling of LECs was induced by tamoxifen treatment at 6 weeks of age. Note dynamic actin remodeling and ruffling specifically at the edges of LEC lobes, most notable at the border between labelled LifeAct-EGFP-expressing and unlabeled LECs. Single plane. Scale bar: 50 µm, total length 7:31 min:sec, speed 1.75 sec/frame.

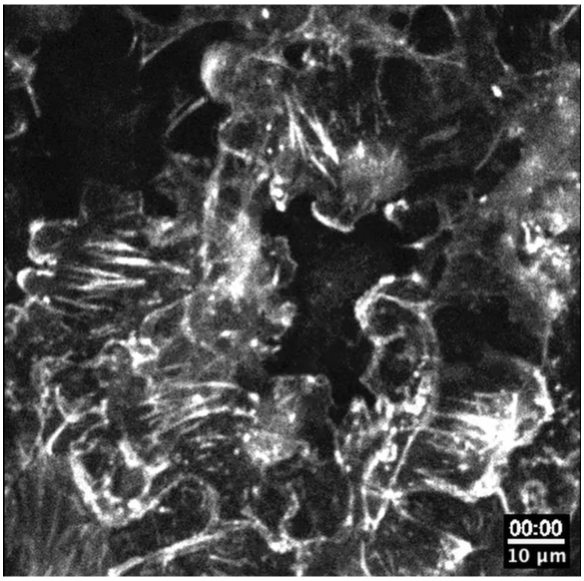

Supplementary Movie 4. Intravital imaging of Lifeact-EGFP showing dynamic actin remodeling in capillary LECs. Higher magnification of **Supplementary Video 3** highlighting a region with highly dynamic actin and prominent remodelling of lobe border (lower left quadrant). Single plane. Scale bar: 10 µm, total length 7:31 min:sec, speed 1.75 sec/frame.

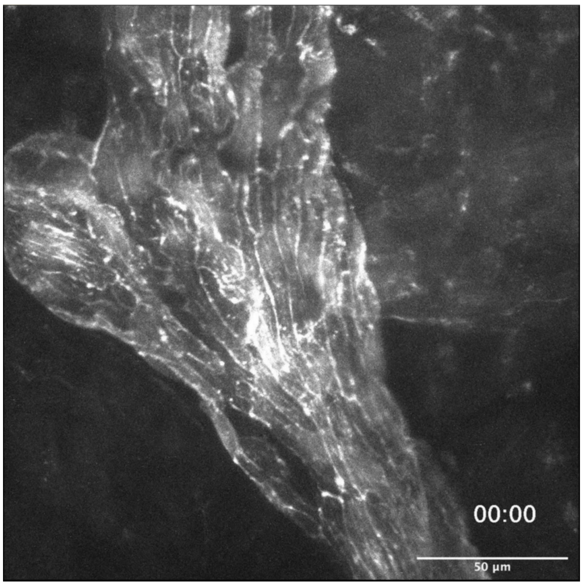

Supplementary Video 5. Intravital imaging of Lifeact-EGFP showing limited actin remodeling in collecting vessel LECs. Real-time intravital two-photon imaging of a dermal collecting lymphatic vessel at the area of a valve in a 9-week-old *LifeAct-EGFP; Vegfr3-CreER^{T2}* BL6-albino mouse. Labelling of LECs was induced by tamoxifen treatment at 6 weeks of age. Note the presence of stable cortical actin and linear radial stress fibers aligned along the vessel axis. Despite vessel contractions induced by lymphatic smooth muscle cells (not labeled), no noticeable remodelling of cortical actin or actin fibers is observed. Maximum projection of a Z-stack. Scale bar: 50 µm, total length 14:31 min:sec, speed 32.29 sec/frame.

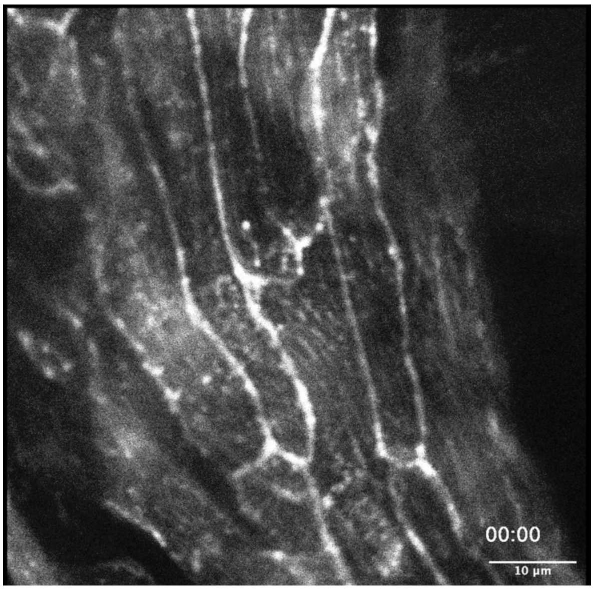

Supplementary Video 6. Intravital imaging of Lifeact-EGFP showing limited actin remodeling in collecting vessel LECs. Higher magnification of **Supplementary Video 5** (central region) imaged at a higher acquisition speed, highlighting the stability of cortical actin despite vessel contractions. Single plane. Scale bar: 10 µm, total length 4:00 min:sec, speed 2.56 sec/frame.

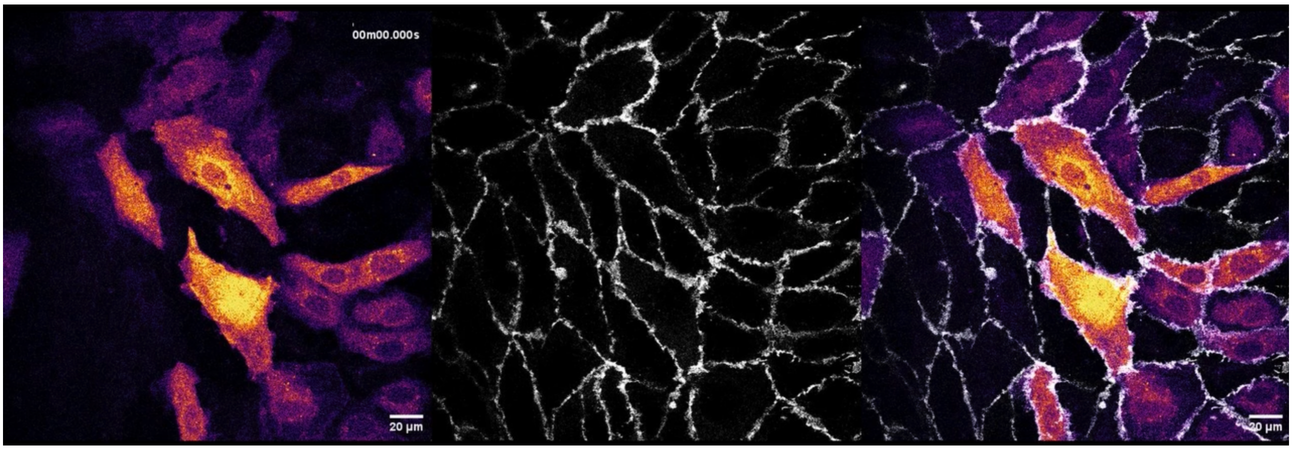

Supplementary Video 7. Time lapse imaging of primary human LECs after optogenetic activation of CDC42. Real time confocal imaging of primary human dermal LECs expressing Lck-mTurquoise2-iLID and SspB-HaloTag-ITSN1(DHPH) (OptoITSN + iLid). Central channel shows localization of VE-cadherin by using a fluorescent conjugated-non blocking antibody. Local photoactivation is induced using 488 nm laser line and is shown in blue squared box. Note the extension of lamellipodia beyond VE-cadherin junctional borders specifically in the area of photo-activation. Scale bar: 20 µm, total length 1:23 min:sec, speed 2.59 sec/frame.

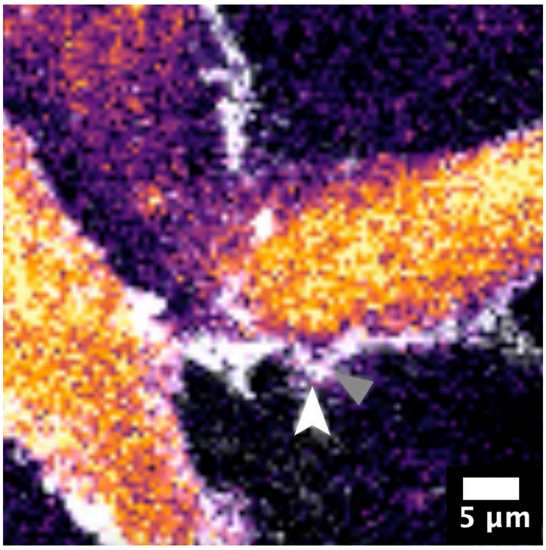

Supplementary Video 8. Time lapse imaging of primary human LECs after optogenetic activation of CDC42. Higher magnification of **Supplementary Video 7.** Real time confocal imaging of primary human dermal LECs expressing Lck-mTurquoise2-iLID and SspB-HaloTag-ITSN1(DHPH) (OptoITSN + iLid). Local photoactivation is induced using 488 nm laser line and is shown by blue squared bar (upper right). Note the extension of lamellipodia (white arrowhead) beyond VE-cadherin junctional borders (grey arrowhead). Scale bar: 5 µm, total length 1:23 min:sec, speed 2.59 sec/frame.

Supplementary References

57. Achard, C. & Aynaud, M. Recherches sur l'impregnation histologique de l'endothelium. *Arch Med Exp* **19**, 437–450 (1907).

58. Gottlob, R. & Hoff, H. F. Histochemical investigations on the nature of large blood vessel endothelial and medial argyrophilic lines and on the mechanism of silver staining. *Histochem. Histochem. Histochim.* **13**, 70–83 (1968).

59. Zand, T., Underwood, J. M., Nunnari, J. J., Majno, G. & Joris, I. Endothelium and 'silver lines'. An electron microscopic study. *Virchows Arch. A Pathol. Anat. Histol.* **395**, 133–144 (1982).

60. Hirata, A., Baluk, P., Fujiwara, T. & McDonald, D. M. Location of focal silver staining at endothelial gaps in inflamed venules examined by scanning electron microscopy. *Am. J. Physiol.* **269**, L403-418 (1995).

61. Zhang, Y. *et al.* Molecular insights into the complex mechanics of plant epidermal cell walls. *Science* **372**, 706–711 (2021).

62. Sabine, A. *et al.* FOXC2 and fluid shear stress stabilize postnatal lymphatic vasculature. *J. Clin. Invest.* **125**, 3861–3877 (2015).

63. Miteva, D. O. *et al.* Transmural flow modulates cell and fluid transport functions of lymphatic endothelium. *Circ. Res.* **106**, 920–931 (2010).

64. Riedl, J. *et al.* Lifeact: a versatile marker to visualize F-actin. *Nat. Methods* **5**, 605–607 (2008).

65. Martinez-Corral, I. *et al*. Blockade of VEGF-C signaling inhibits lymphatic malformations driven by oncogenic PIK3CA mutation. *Nat. Commun.* **11**, 2869 (2020).

66. Corliss, B. A. *et al.* REAVER: A program for improved analysis of high-resolution vascular network images. *Microcirc. N. Y. N 1994* **27**, e12618 (2020).

67. Otani, T., Ichii, T., Aono, S. & Takeichi, M. Cdc42 GEF Tuba regulates the junctional configuration of simple epithelial cells. *J. Cell Biol.* **175**, 135–146 (2006).

68. Schürmann, S. *et al.* The IsoStretcher: An isotropic cell stretch device to study mechanical biosensor pathways in living cells. *Biosens. Bioelectron.* **81**, 363–372 (2016).

69. Schnelle, M. *et al.* Echocardiographic evaluation of diastolic function in mouse models of heart disease. *J. Mol. Cell. Cardiol.* **114**, 20–28 (2018).
