# Supplementary material for: Dynamic cytoskeletal regulation of cell shape supports resilience of lymphatic endothelium
Source: Nature. 2025 Mar 19;641(8062):465–75. doi: 10.1038/s41586-025-08724-6 (PMC12058511; doi:10.1038/s41586-025-08724-6)
Supplement: Supplementary file 2 — Reporting Summary [file 41586_2025_8724_MOESM2_ESM.pdf]

nature portfolio

Corresponding author(s): Taija Mäkinen

Last updated by author(s): 12/04/2024

Reporting Summary

Nature Portfolio wishes to improve the reproducibility of the work that we publish. This form provides structure for consistency and transparency in reporting. For further information on Nature Portfolio policies, see our Editorial Policies and the Editorial Policy Checklist.

Statistics

For all statistical analyses, confirm that the following items are present in the figure legend, table legend, main text, or Methods section.

n/a	Confirmed	
☐	☒	The exact sample size (n) for each experimental group/condition, given as a discrete number and unit of measurement
☐	☒	A statement on whether measurements were taken from distinct samples or whether the same sample was measured repeatedly
☐	☒	The statistical test(s) used AND whether they are one- or two-sided<br>*Only common tests should be described solely by name; describe more complex techniques in the Methods section.*
☒	☐	A description of all covariates tested
☐	☒	A description of any assumptions or corrections, such as tests of normality and adjustment for multiple comparisons
☐	☒	A full description of the statistical parameters including central tendency (e.g. means) or other basic estimates (e.g. regression coefficient) AND variation (e.g. standard deviation) or associated estimates of uncertainty (e.g. confidence intervals)
☐	☒	For null hypothesis testing, the test statistic (e.g. F, t, r) with confidence intervals, effect sizes, degrees of freedom and P value noted<br>*Give P values as exact values whenever suitable.*
☒	☐	For Bayesian analysis, information on the choice of priors and Markov chain Monte Carlo settings
☒	☐	For hierarchical and complex designs, identification of the appropriate level for tests and full reporting of outcomes
☒	☐	Estimates of effect sizes (e.g. Cohen's d, Pearson's r), indicating how they were calculated

Our web collection on statistics for biologists contains articles on many of the points above.

Software and code

Policy information about availability of computer code

Data collection	Leica Application Suite (Version 4.5.0 25531 and earlier) [image aquisition]<br>BD FACSDiva Software (Version 8.0) (BD Biosciences) [flow cytometry]
Data analysis	Image J (Version 2.9.0/1.53t or earlier)<br>Adobe Photoshop (Version 27.3)<br>Huygens Essential software (v 19.04) (Scientific Volume Imaging) [image deconvolution]<br>Graphpad Prism 9.0<br>MATLAB R2020a<br>FlowJo 10.5.0-10.5.3 (TreeStar) [flow cytometry]<br>MorphoDynamX/MorphoMechanX version 2.0 revision: 2-1459<br>Code for Finite element method (FEM) simulations is available in Zenodo with the identifier doi: 10.5281/zenodo.13880404

For manuscripts utilizing custom algorithms or software that are central to the research but not yet described in published literature, software must be made available to editors and reviewers. We strongly encourage code deposition in a community repository (e.g. GitHub). See the Nature Portfolio guidelines for submitting code & software for further information.

Data

Policy information about availability of data

All manuscripts must include a data availability statement. This statement should provide the following information, where applicable:

- Accession codes, unique identifiers, or web links for publicly available datasets
- A description of any restrictions on data availability
- For clinical datasets or third party data, please ensure that the statement adheres to our policy

All source data supporting the quantitative findings of this study are provided as a Source Data file. Images of annotated lymphatic capillary junctions in mouse ear skin used for Fig. 1g are publicly available in Zenodo with the identifier doi: 10.5281/zenodo.13880404. All other data supporting the findings are available within the paper and its supplementary information files.

Research involving human participants, their data, or biological material

Policy information about studies with human participants or human data. See also policy information about sex, gender (identity/presentation), and sexual orientation and race, ethnicity and racism.

Reporting on sex and gender	n/a
Reporting on race, ethnicity, or other socially relevant groupings	n/a
Population characteristics	n/a
Recruitment	n/a
Ethics oversight	n/a

Note that full information on the approval of the study protocol must also be provided in the manuscript.

Field-specific reporting

Please select the one below that is the best fit for your research. If you are not sure, read the appropriate sections before making your selection.

☒ Life sciences ☐ Behavioural & social sciences ☐ Ecological, evolutionary & environmental sciences

For a reference copy of the document with all sections, see nature.com/documents/nr-reporting-summary-flat.pdf

Life sciences study design

All studies must disclose on these points even when the disclosure is negative.

Sample size	No statistical methods were used to pre-determine sample size.<br>For in vivo experiments, a minimum of 3 mice per condition was used, except for Fig. 4c and d, n=2 for 9 week-old mice.<br>For in vitro experiments a minimum of 3 biological replicates were used, except for ED Fig. 10a (validation of integrin beta1 inhibition), n=1-2 stretch holders.<br>The sample size of 3 was chosen as the minimum required to perform statistical tests.
Data exclusions	No data were excluded.
Replication	All data has been successfully replicated in at least two independent experiments.
Randomization	Allocation of mice into experimental groups was based on genotype. Littermate controls were included. Both female and male mice were included in analyses. Data were collected from different litters on different days and experiments were performed for different batches at different time points.<br>For in vitro experiments, allocation into experimental groups was performed randomly. Most biological replicates were analysed independently in different batches at different time points.
Blinding	No blinding was done in the data collection, analysis and quantifications. Quantification of LEC and vessel parameters (cell size, lymphatic vessel diameter, LEC overlap) was done in an unbiased automated fashion using ImageJ or the MATLAB script "REAVER" (Corliss, B. A. et al. Microcirc. N. Y. N 1994 27, e12618 (2020).

Reporting for specific materials, systems and methods

We require information from authors about some types of materials, experimental systems and methods used in many studies. Here, indicate whether each material, system or method listed is relevant to your study. If you are not sure if a list item applies to your research, read the appropriate section before selecting a response.

Materials & experimental systems

n/a	Involved in the study
☐	☒ Antibodies
☐	☒ Eukaryotic cell lines
☒	☐ Palaeontology and archaeology
☐	☒ Animals and other organisms
☒	☐ Clinical data
☒	☐ Dual use research of concern
☒	☐ Plants

Methods

n/a	Involved in the study
☒	☐ ChIP-seq
☐	☒ Flow cytometry
☒	☐ MRI-based neuroimaging

Antibodies

Antibodies used

The following antibodies were used for whole mount immunofluorescence (dilution 1:100-1:500): chicken anti-GFP (ab13970, Abcam), goat anti-mouse VEGFR3 (AF743, R&D Systems), goat anti-mouse VE-cadherin (R&D Systems, AF1002), goat anti-mouse PECAM1 (R&D, Systems AF3628), mouse anti-HA tag, Alexa Fluor 647 (Cell Signalling Technology, 6E2), rabbit anti-alpha tubulin (Abcam, ab52866), rabbit anti-GFP (A11122, Thermo Fisher Scientific), rabbit anti-DsRed (Takara Bio, 632496), rabbit anti-mouse LYVE1 (Reliatech, 103-PA50AG), rabbit anti-mouse CLDN5 (Invitrogen, 34-1600), rat anti-mouse PECAM1 (553370, BD Pharmingen), rat anti-mouse LYVE1-Alexa Fluor™ 488, Clone ALY7 (Invitrogen, 53-0443-82), rat anti-mouse LYVE1 (R&D Systems (MAB2125), rat Anti-Mouse CD29 Clone 9EG7 (BD Pharmingen, 553715) All secondary antibodies were conjugated to Cy3(JIR, 712-165-153),(JIR, 711-166-152), Dylight 405 (JIR, 712-475-153), Alexa Fluor 488 (JIR, 703-545-155), (JIR, 712-545-153), (JIR, 711-545-152), Alexa Fluor 594 (JIR, 705-585-147), Alexa Fluor 647(JIR, 712-605-153),(JIR, 705-605-147),(JIR, 711-605-152) or Alexa Fluor 680 (JIR, 705-625-147) were raised in donkey and obtained from Jackson ImmunoResearch(JIR) . Secondary antibodies conjugated to AF405+ (# A48268), AF488+(# A48269), AF555+ (# A32794) or AF647+ (# A32849)were obtained from ThermoFisher Scientific.

The following antibodies or reagents were used for immunostaining of cells: DAPI (MBD0015-1ML, Sigma Aldrich), goat anti-mouse VE-cadherin (AF1002, R&D Systems), mouse anti-human PECAM1 (clone JC70A, M0823, Dako), mouse anti-Integrin β1 Antibody, activated, clone HUTS-4 (EMD Millipore, MAB2079Z), mouse Integrin β1/ITGB1 Antibody (TS2/16) Alexa Fluor 488 (Santa Cruz, sc-53711 AF488), rabbit Phospho-Myosin Light Chain 2 (Cell Signalling Technology, 3671), rat anti-Human CD29, Clone Mab 13 (BD Pharmingen, 552828) SPY-555 actin (Spirochrome). All secondary antibodies were conjugated to Cy3(JIR, 712-165-153),(JIR, 711-166-152), Dylight 405 (JIR, 712-475-153), Alexa Fluor 488 (JIR, 715-545-151), (JIR, 703-545-155), (JIR, 712-545-153), (JIR, 711-545-152), Alexa Fluor 594 (JIR, 705-585-147), Alexa Fluor 647(JIR, 712-605-153),(JIR, 705-605-147),(JIR, 711-605-152) were raised in donkey and obtained from Jackson ImmunoResearch(JIR) .

The following antibodies or reagents were used for western blot analysis of cell lysates: rabbit anti-CDC42, Clone 11A11 (Cell Signalling Technology, 2466), rabbit anti-human GAPDH, Clone 14C10 (Cell Signalling Technology, 2118). Secondary antibodies conjugated to HRP were obtained from Jackson ImmunoResearch.

For following antibodies were used for FACS: rat anti-mouse CD16/CD32 (eBioscience 14-0161-85), PDPN (8.1.1, PE, eBioscience 12-5381-81), CD31/PECAM1 (390, PE-Cyanine7, eBioscience 25-0311-82), CD45 (30-F11, eFluor 450, eBioscience 48-0451-82), CD11b (M1/70, eFluor 450, eBioscience 48-0112-82), Ki67 (SolA15, eFluor 660, eBioscience 50-5698-80).

Validation

The antibodies used in this study were validated for the species and applications by the manufacturers. They have all been used in previous publications by us and/or others.

Antibodies used for immunostaining:
chicken anti-GFP: Abcam provides several references for validation. https://www.abcam.com/gfp-antibody-ab13970.html
goat anti-mouse VEGFR3: R&D Systems provides several references for validation. https://www.rndsystems.com/products/mouse-vegfr3-flt-4-antibody_af743
goat anti-mouse VE-cadherin: R&D Systems provides several references for validation, and additionally this antibody was validated in this study by using of VE-cadherin-GFP reporter mice. https://www.rndsystems.com/products/mouse-ve-cadherin-antibody_af1002
goat anti-mouse PECAM1: R&D Systems provides several references for validation. https://www.rndsystems.com/products/mouse-rat-cd31-pecam-1-antibody_af3628
mouse anti-HA tag, Alexa Fluor 647: Cell Signalling Technology provides several references for validation. https://www.cellsignal.com/products/antibody-conjugates/ha-tag-6e2-mouse-mab-alexa-fluor-647-conjugate/3444
rabbit anti-alpha tubulin: Abcam provides provides several references for validation. https://www.abcam.com/products/primary-antibodies/alpha-tubulin-antibody-ep1332y-microtubule-marker-ab52866.html
rabbit anti-GFP: Thermo Fisher Scientific provides several references for validation. https://www.thermofisher.com/antibody/product/GFP-Antibody-Polyclonal/A-11122
rabbit anti-mouse CLDN5: Thermo Fisher Scientific provides several refernces for validation.
https://www.thermofisher.com/antibody/product/Claudin-5-Antibody-Polyclonal/34-1600
rabbit anti-DsRed: TRakara Bio provides several references for validation. https://www.takarabio.com/products/antibodies-and-elisa/fluorescent-protein-antibodies/red-fluorescent-protein-antibodies
rabbit anti-mouse LYVE1: Reliatech provides several references for validation. https://www.reliatech.de/products/antibodies/polyclonal-antibodies/product/103-pa50ag/
rat anti-mouse PECAM1: BectonDickinson provides several references for validation. https://www.bdbiosciences.com/eu/applications/research/stem-cell-research/cancer-research/mouse/purified-rat-anti-mouse-cd31-mec-133/p/553370

rat anti-mouse LYVE1: R&D Systems provides several references for validation.https://www.rndsystems.com/products/mouse-lyve-1-antibody-223322_mab2125
rat anti-mouse LYVE1-Alexa Fluor™ 488, Clone ALY7: Invitrogen provides several references for validation. https://www.thermofisher.com/antibody/product/LYVE1-Antibody-clone-ALY7-Monoclonal/53-0443-82
rat anti-mouse CD29, Clone 9EG7 (RUO): BD Pharmingen provides several references for validation. https://www.bdbiosciences.com/en-eu/products/reagents/flow-cytometry-reagents/research-reagents/single-color-antibodies-ruo/purified-rat-anti-mouse-cd29.553715

Antibodies used for immunostaining of cells:
goat anti-mouse VE-cadherin: R&D Systems provides several references for validation. https://www.rndsystems.com/products/mouse-ve-cadherin-antibody_af1002?gclid=Cj0KCQjw2qKmBhCfARIsAFy8buJHxptyt01pulJtf9L724wOf2ropFHp8poOiSS3BphyRhTa2fTmvi8aAt2KEALw_wcB&gclsrc=aw.ds
mouse anti-human PECAM1: Agilent Dako provides several references for validation. https://www.agilent.com/en/product/immunohistochemistry/antibodies-controls/primary-antibodies/cd31-endothelial-cell-%28dako-omnis%29-76224
mouse anti-Integrin β1 Antibody, activated, clone HUTS-4 EMD: Millipore provides several references for validation. https://www.merckmillipore.com/SE/en/product/Anti-Integrin-1-Antibody-activated-clone-HUTS-4-Azide-Free,MM_NF-MAB2079Z
mouse Integrin β1/ITGB1 Antibody (TS2/16), Alexa Fluor 488: Santa Cruz provides several references for validation. https://www.scbt.com/p/integrin-beta1-antibody-ts2-16
rabbit Phospho-Myosin Light Chain 2: Cell Signalling Technology provides several references for validation. https://www.cellsignal.com/products/primary-antibodies/phospho-myosin-light-chain-2-ser19-antibody/3671
rat anti-Human CD29, Clone Mab 13: BD Pharmingen provides several references for validation. https://www.bdbiosciences.com/en-se/products/reagents/flow-cytometry-reagents/research-reagents/single-color-antibodies-ruo/purified-rat-anti-human-cd29.552828

Antibodies used for western blot analysis of cell lysates:
rabbit anti-CDC42, Clone 11A11: Cell Signalling Technology provides several references for validation. https://www.cellsignal.com/products/primary-antibodies/cdc42-11a11-rabbit-mab/2466
rabbit anti-human GAPDH, Clone 14C10: Cell Signalling Technology provides several references for validation. https://www.cellsignal.com/products/primary-antibodies/gapdh-14c10-rabbit-mab/2118

Antibodies used for flow cytometry:
rat anti-mouse CD16/CD32: eBioscience provides several references for validation. https://www.thermofisher.com/antibody/product/CD16-CD32-Antibody-clone-93-Monoclonal/14-0161-82

Eukaryotic cell lines

Policy information about cell lines and Sex and Gender in Research

Cell line source(s)	Primary human dermal lymphatic endothelial cells (HDLEC) from juvenile foreskin (C-12216, PromoCell), human umbilical vein endothelial cells (C-12200, PromoCell), HEK293T cells (CRL-3216, American Tissue Culture Collection; Manassas, VA, USA)
Authentication	Authenticated by supplier and verified based on morphology (HEK293T) or immunohistochemistry profile (HDLEC)
Mycoplasma contamination	Cells were tested negative for mycoplasma contamination
Commonly misidentified lines (See ICLAC register)	No commonly misidentified lines were used in this study

Animals and other research organisms

Policy information about studies involving animals; ARRIVE guidelines recommended for reporting animal research, and Sex and Gender in Research

Laboratory animals	Tie2-Cre (Koni et al, 2001), Vegfr3-CreERT2 (Martinez-Corral et al, 2016), Prox1-CreERT2 (Bazigou et al, 2011), iMb2-Mosaic (Pontes-Quero et al, 2017), Cdh5-GFP (encoding VE-cadherin-GFP fusion protein) (Winderlich et al, 2009), Cldn5flox (Frye et al, 2020), Cdc42flox (Wu et al, 2006) and Itgb1flox (Raghavan et al, 2000) were analyzed on a C57BL/6J background, with the exception of the iMb2-Mosaic;Vegfr3-CreERT2 mice used for intravital imaging experiments that were crossed to a C57BL/6-albino (B6(Cg)-Tyrc-2J/J) background. R26-LifeAct-EGFP mice were generated as described in the manuscript. Both female and male mice were used for analysis and no differences in the phenotype between the sexes were observed. Both embryonic (E17) and postnatal (up to 41 weeks of age) were used for experiments. The stage/age is stated in the figures and/or legends. Mice were housed in individually ventilated cages (GM500, Tecniplast) under a 12:12-h dark–light cycle (light from 07:00 to 19:00) at 22 ± 1°C under 40-60% humidity with ad libitum access to food and water.
Wild animals	The study did not involve wild animals.
Reporting on sex	Both female and male mice were included in analyses. No differences in the phenotype between the sexes were observed.
Field-collected samples	The study did not involve samples collected from the field.
Ethics oversight	All experimental procedures were approved by the Uppsala Laboratory Animal Ethical Committee, Sweden, or the National Animal Experiment Board in Finland.

Note that full information on the approval of the study protocol must also be provided in the manuscript.

Flow Cytometry

Plots

Confirm that:

☐ The axis labels state the marker and fluorochrome used (e.g. CD4-FITC).

☐ The axis scales are clearly visible. Include numbers along axes only for bottom left plot of group (a 'group' is an analysis of identical markers).

☐ All plots are contour plots with outliers or pseudocolor plots.

☐ A numerical value for number of cells or percentage (with statistics) is provided.

Methodology

Sample preparation	For FACS analysis of proliferating cells ear skins were dissected, cut into small pieces and digested in Collagenase IV (Life Technologies) 10 mg/ml, DNase1 (Roche) 0.1 mg/ml and FBS 0.5 % (Life Technologies) in PBS at 37 °C for 30 min. Collagenase activity was quenched by dilution with FACS buffer (PBS, 0.5 % FBS, 2 mM EDTA) and digestion products were filtered twice through 70 µm nylon filters (BD Biosciences). Cells were washed with FACS buffer and immediately processed for immunostaining first by blocking Fc receptor binding with rat anti-mouse CD16/CD32 followed by incubation with antibodies targeting PDPN, CD31/PECAM1, CD45 and CD11b. After staining, cells were washed with PBS and then stained for dead cells using the blue LIVE/DEAD® fixable dead cell stain kit (Life Technologies), followed by fixation and permeabilization using the Foxp3/Transcription factor staining kit according to the manufacturer's instructions. Finally cells were incubated with rat serum and KI67 antibody.
Instrument	Cells were analyzed on a BD LSR Fortessa cell analyzer equipped with 5 lasers (355, 405, 488, 561 and 643 nm), or CytoFLEX Flow Cytometer (Beckman Coulter) with 4 lasers (405, 488, 561 and 633 nm).
Software	FlowJo software version 10.5.0-10.5.3 (TreeStar)
Cell population abundance	Analysis of proliferating LECs: ECs (PECAM1+) of total cells 1.5-2%; LECs (PDPN+) of ECs 20-30%, KI67+ LECs of all LECs 1-60%.
Gating strategy	Single viable cells were gated from FSC-A/SSC-A, FSC-H/FSC-W and SSC-H/SSC-W plots followed by exclusion of dead cells in the UV dump channel. FMO controls were used to set up the subsequent gating scheme to obtain cell populations and quantification of proliferating cells. Gating was done as described and exemplified in Martinez-Corral et al, Nat Commun 2020.

☐ Tick this box to confirm that a figure exemplifying the gating strategy is provided in the Supplementary Information.
